# Analysing the acute toxicity of e-cigarette liquids and their vapour on human lung epithelial (A549) cells *in vitro*

**DOI:** 10.1016/j.toxrep.2025.102092

**Published:** 2025-07-18

**Authors:** Fern Findlay-Greene, Samantha Donnellan, Sharron Vass

**Affiliations:** Centre for Biomedicine and Global Health, School of Applied Sciences, Edinburgh Napier University, Sighthill Court, Edinburgh, Scotland EH11 4BN, UK

**Keywords:** E-cigarettes, ENDS, Vaping, Cytotoxicity, Lung cells, Secondary vapour, Vaping flavourings

## Abstract

The use of alternative tobacco products such as vaping devices has significantly increased over the last 5-years, with the largest increase being amongst 18–25-year-olds. While the quantity of nicotine is tightly regulated, the composition of e-liquid flavourings is largely unregulated, and often absent from product labels. Herein, we compare the toxicity of carrier liquids propylene glycol (PG) & vegetable glycerine (VG) with five popular flavour concentrates: menthol, cherry, butterscotch, vanilla bourbon and tobacco on human alveolar type II cell-like A549 cells. The flavourings were tested in both liquid and vapour form and a vapour assay was developed to assess cytotoxicity of the flavourings. Our results conclude that menthol liquid was the most cytotoxic (LD_50_ = <0.5 % over a <4 h exposure). Followed by cherry and vanilla bourbon which elicited a similar response at 4 % over 8 h exposure. Tobacco only reached 50 % toxicity at a concentration > 4 % over 24 h exposure. Butterscotch displayed similar toxicity profiles to PG and VG where cytotoxicity exceeded 20 % at 8 % concentration at all time points. The cytotoxicity of menthol was further evaluated as a vapour, with a significant reduction in viable cells and a 5-fold increase in the number of necrotic cells with only 11 % of cells remaining viable after 5 vaping episodes. Analysis revealed the presence of toxic chemicals and heavy metals in the fluids therefore further research is required to fully elucidate the long-term usage of flavourings with vaping devices and the impact this may have on human lung health.

## Highlights

• Vaping fluid flavourings were found to contain potentially carcinogenic chemicals and heavy metals.
• A method to model vaping and secondary exposure to vapour was devised.
• *In vitro* cytotoxicity of vaping flavourings in the absence of nicotine.
• Menthol was the most cytotoxic flavouring tested, and tobacco was the least cytotoxic.
• Cell viability is significantly reduced when exposed to aerosolised menthol vapour from an electronic nicotine delivery device.

## 1 Introduction

The use of alternative tobacco products, such as electronic nicotine delivery systems (ENDS) and vaping devices has dramatically increased since their inception in 2003. In the UK alone, the estimated number of users reached 5.6 M in 2024, with the most significant growth seen in the 18–25 age group, including individuals who had not previously used tobacco products [1], [2]. This surge coincides with the introduction of more versatile devices, including customisable “box-mods” and disposable systems [3]. According to the Global State of Tobacco Harm Reduction (GSTHR) the number of ENDS users globally has risen from 68 M in 2020–114 M in 2023 [4], [5]. The e-cigarette market is projected to more than double from $17 billion in 2022 to $40.6 billion by 2030, with the majority of current market in the United States, United Kingdom, Europe, China and Japan [6].

Initially promoted as a safer alternative to tobacco smoking, ENDs are often viewed as a less hazardous alternative. Indeed, reviews undertaken by Public Health England concluded that “*vaping was 95 % less harmful to smoking”* [7], [8]. Furthermore, a 2025 Cochrane review concluded that for every 100 people using e-cigarettes 9 % might stop smoking compared to 6 % of people using nicotine-replacement therapy [9]. *S*ince cigarettes cause harm by the combustion of tobacco which releases a wide range of toxic chemicals including tar and carbon monoxide, e-cigarettes are thought to be less harmful as they deliver nicotine by creating a vapour. However, concerns have been raised regarding the toxicity of vaping fluids [10] and studies have shown that some commercially available devices produce vapours containing condensed hydrocarbon-like-compounds typically associated with combustion [11], [12].

Vaping fluids are composed of carrier liquids and flavourings and can be prepared with or without nicotine. The carrier liquids are propylene glycol (PG) and vegetable glycerine (VG) which can be combined in different ratios, depending on user preference. PG and VG are deemed to have low toxicity when ingested [13], [14] and when free from contaminants or flavourings, the liquids have been shown to cause no significant impact on lung epithelial cells [15], [16], [17], or pulmonary function in healthy or asthmatic human test subjects [18].

Flavourings can be purchased pre-mixed or as individual components, allowing the user to create their own blends. The latest FDA figures show that 87.6 % of youth users choose the fruity, dessert and menthol flavours [19]. The chemical composition of e-liquids is often omitted from labelling, but common flavourings used in e-liquids may include, isoamyl acetate (pear or banana), benzaldehyde (bitter almond or cherry), cinnamaldehyde (cinnamon), ethyl maltol (caramelised sugar / candy floss) or ethyl vanillin (vanilla) [20]. Although these flavourings and the carrier liquids PG and VG are generally recognised as safe (GRAS) when ingested as food [21], the vaporising effect at high temperatures and long-term health risks are yet to be fully elucidated.

The temperature of the heating coils within vaping devices are known to be highly variable [22], and comparable to pyrolysis conditions (i.e. heating an organic material in high temperatures) which can change the chemical composition of a vaping mixture [23]. It has been documented that volatile carbonyls such as formaldehyde, acrolein, and acetaldehyde are present as by-products of the ENDS vaporising process [24], [25], [26]. A study by Jenson *et al*., (2015) reported that the levels of formaldehyde emitted from e-cigarettes was 5–15 fold higher than tobacco cigarettes, but that this was dependent on the voltage of the e-cigarette vaporiser being at the highest setting (5 V) [27]. A replica study conducted by Farsalinos *et al.*, (2017) concluded that using voltages above 4.2 created a ‘dry-puff’ phenomena, which although producing higher levels of formaldehyde, resulted in an unpleasant taste to the user, so would not mimic realistic conditions [28]. The latest generation of devices tend to have a nominal voltage of between 3.3 and 4.2 V, [29], and are less likely to generate formaldehyde through excessive heating, however, a recent study found that four of sixteen e-liquid products contained formaldehyde even before vaporisation [30] highlighting the importance of analysing vaping flavourings.

As the vaping market rapidly expanded there was little regulation over quality control and handling of flavouring products. Regulations under the Tobacco Products Directive (TPD) were introduced in the UK in 2016. This directive prohibited the advertising of vaping products, limited the concentration of nicotine they could contain (20 mg/mL), and limited the quantity of e-liquid that could be bought (2 mL per cartridge, or 10 mL for refills). Although the amount of nicotine is regulated in the UK, this is not the case elsewhere. There are an increasing number of counterfeit products infiltrating the UK market, and one study found nicotine in 81.3 % of counterfeit refill fluids that were labelled to contain 0 mg/mL [31]. Furthermore, there is limited regulation or safety information surrounding specific refill flavourings and scarce information about the potential effects these might have on the environment when disposed of or accidentally released. Studies have demonstrated that the cytotoxicity of flavourings is highly variable [32], and different batches of the same product have been documented to contain different chemical compositions [33], [34], again emphasising the need for tighter regulations on these products and for more research into their potential health effects.

There is a growing body of evidence regarding the safety of vaping fluids, particularly regarding the presence of potentially harmful substances and their adverse effects on respiratory, cardiovascular and neurological systems [35], [36], [37]. Studies have shown that the carrier liquids PG and VG can induce oxidative stress and inflammatory responses, especially when thermally degraded [38], [39], [40], [41].

It has been documented that the vaping fluids used in e-cigarettes can induce cytotoxic effects such as oxidative stress, inflammation, and DNA damage, even in the absence of nicotine [41], [42], [43]. Recent data also highlight concerns over cardiovascular and pulmonary risks including endothelia dysfunction, and increased susceptibility to inflammatory conditions such as bronchiolitis obliterans, often referred to as ‘popcorn-lung’ [44], [45], [46].

Although the UK banned diacetyl in e-liquids in 2016 under the TPD regulations, studies have detected trace amount of diacetyl and structurally similar compounds (e.g., 2,3-pentanedione and acetoin) in e-cigarette aerosols and refill fluids, particularly in dessert and sweet-flavoured products [47], [48], [49]. This is of concern because acetoin, often used as a diacetyl substitute, has been shown to convert to diacetyl under particular heating or storage conditions for example longer than 18-months [50].

Existing research has been conducted *in vitro, in vivo* and using human test subjects, however there are disparities between model systems used (immortalised cell lines, animal models), the mode of delivery (liquid, vapour), and the methods of analysis (cytotoxicity, oxidative stress, and inflammatory markers). Although there are guidelines for evaluating the toxicity of e-liquids recommended by the Organisation for Standardisation (ISO) and the Cooperation Centre for Scientific Research Relative to Tobacco (CORESTA) which include parameters on puff topography such as duration, volume and interval, this does not consider the rapidly evolving e-cigarette market, and the inter-individual puffing variability [51].

The objective of this study was to evaluate the toxicological effects of five nicotine free e-cigarette flavourings sold in the UK - tobacco, butterscotch, vanilla bourbon, cherry, and menthol and the carrier liquids PG and VG on human lung epithelial cells. This analysis was done by assessing cellular cytotoxicity via membrane integrity and key apoptotic markers and stages. Additionally, we used qualitative Gas Chromatography-Mass Spectrometry (GC/MS) and Atomic Emission Spectroscopy (ACP/AES) to characterise the chemical composition of the flavourings to correlate chemical constituents with observed biological responses. This study adds to the body of evidence characterising the toxicity of flavourings (particularly that of menthol) and can be used by policy makers to enhance product labelling legislation thereby aiding consumers to make informed choices.

## 2 Methods

### 2.1 Cell culture

A549 cells (Sigma Aldrich) were grown in RPMI (Gibco, UK) supplemented with 10 % FBS (Gibco), 1 % L-Glutamate (Gibco) and 1 % Penicillin Streptomycin (pen strep) (Gibco) at 37˚C, 5 % CO_2_. All experiments were performed with A549 cells cultured in RPMI. Cells were periodically tested for the presence of mycoplasma using a PCR-based Venor™ GeM Mycoplasma Detection Kit.

### 2.2 Test substances and liquid exposure mode

Vaping fluids (vegetable glycerine [VG], propylene glycol [PG], tobacco, butterscotch, vanilla bourbon, cherry, and menthol) were all purchased from DarkStar® Vapour (UK). The five flavourings chosen as these represent the five most popular categories of flavouring sold in the UK according to ECigIntelligence [52]. The vaping fluids were diluted in RPMI supplemented with 10 % FBS, 1 % L-glutamate and 1 % pen strep to the following concentrations: 0, 0.125, 0.25, 0.5, 1.0, 2.0, 4.0, 6.0 and 8.0 %. Triton-X at 0.25 % was used as a positive control and media with cells only was used as a negative control. Each concentration was vortexed for 20 s prior to addition to the pre-washed cells.

### 2.3 Cell viability

A549 cells were seeded at 1 × 10^5^ per well onto a 12 well plate and left for 24 h at 37°C, 5 % CO_2_ to attach and form a monolayer representative of the lung epithelium. The epithelial cells were exposed to the different concentrations of vaping fluids over 2, 4, 8, 24 or 48 h and the cell viability was calculated by nigrosin exclusion and confirmed via an automated cell counter using propidium iodide cassettes. The experiments were performed in triplicate and the data presented as percentage cell death for each concentration.

### 2.4 Cytotoxicity testing using Alamar blue™

Cells were seeded at 1 × 10^4^ cells per well onto a 96-well plate and left for 24 h prior to the addition of treatments, as previously described. The cells were then incubated for 2, 4, 8, 24, and 48 h and the assay was performed using the manufacturer’s guide. Briefly, 2 h prior to the required timepoint, Alamar blue™ (Invitrogen) reagent was added to each well (10 µL) and left to incubate at 37°C, 5 % CO_2_. Alamar Blue™ is a resazurin-based solution that functions as a cell health indicator to quantitatively measure viability. The plates were read at 600 nm and 530 nm using a Tecan Sunrise™ plate reader every hour following an initial 1 h incubation for an additional 3 hr. The data presented within is following 2 h incubation with Alamar blue™ at the desired treatment incubation time. The percentage reduction of the reagent was calculated using a formula set out in the manufacturer’s technical guide. The experiments were performed in triplicate and the data presented as percentage viability for each concentration.

### 2.5 Membrane impairment by LDH assay

Cells were seeded at 1 × 10^4^ cells per well onto a 96-well plate and left for 24 h prior to the addition of treatments, as previously described. The cells were then incubated for 4, 8 and 24 h and the assay was performed using the manufacturer’s guide. Briefly, following required incubation periods, 50 µL aliquots were transferred to a fresh 96 well flat clear bottom plate. Cytotox96® (Promega) was added (50 µL) to each sample well and incubated for 30 min at room temperature (RT). The plates were protected from light exposure. Following incubation 50 µL of stop solution was added to each sample well and the absorbance was measured immediately at 490 nm using a Tecan Sunrise™ plate reader. The assay quantifies LDH release as a marker of membrane impairment and the data was presented as percentage cytotoxicity as recommended by the manufacturer’s guide. The experiments were performed in triplicate and the data presented as percentage cytotoxicity for each concentration.

## 3 Determining cell death by FACS analysis

Cells were seeded at 2.5 × 10^5^ cells per well into either a 6-well plate or 35 mm culture dish and incubated overnight at 37°C, 5 % CO_2_ to allow the formation of a monolayer to mimic the lung epithelium before treatments, as previously described. Camptothecin treatment was used as a positive apoptotic control and Triton X 0.25 % was used as a positive necrotic control. The supernatant was collected and centrifuged to obtain any already detached cells and remaining cells were washed with sterile PBS and removed from wells or culture dish using 1X Trypsin (Gibco). Cell solution was neutralised using RPMI supplemented with 10 % FBS, 1 % L-Glutamine, 1 % pen strep and centrifuged at 300XG for 5 min to obtain cell pellet. The annexin/PI staining (Bio Legends) was performed as directed by manufacturers guidelines. Briefly, the cell pellet was washed twice by resuspending cell pellet in wash buffer (Bio Legends) and centrifuging at 300XG for 5 min. The cell pellet was resuspended in Annexin/PI binding buffer (100 µL) provided in the kit and transferred to a FACS tube followed by annexin stain (5 µL) and PI stain (10 µL), the cell suspension was gently mixed and left to incubate at RT in the dark for 15 min. Following incubation, 400 µL of annexin/PI binding buffer was added to each tube and then samples were analysed using a BD FACSCelesta™. The experiments were performed in triplicate.

### 3.1 NFκB translocation

One of the early hallmarks of apoptosis is the translocation of NFκB to the nucleus, [53], therefore cells were stained for NFκB and MitoTracker™. Briefly during cell seeding a 13 mm coverslip was added to each well. MitoTracker™ red (Thermo Fischer Scientific, UK) was added to each well prior to treatments and incubated for 45 min at 37°C, 5 % CO_2_. After incubation MitoTracker™ was removed, cells washed with sterile PBS and the treatments were added as previously described. Following treatments, coverslips were washed 3X in sterile PBS and placed into an immunostaining box. 4 % Paraformaldehyde (50 µL) was added to each coverslip and left to incubate in a fume hood for 15 min to allow the cells to fix to the coverslips. Following this incubation, the coverslips were washed in sterile PBS 3X and then 0.2 % Triton- X (50 µL) was added to each coverslip and incubated at RT for 5 min. A subsequent wash step was then performed (3 X sterile PBS) and 10 % FBS solution was used for blocking for 15 min, followed by another PBS wash [3X]. NFκB p65(A)Rb antibody (SantaCruz, product code Sc-109) was diluted in 1 % FBS at 1:1000 dilution. The primary antibody was then added to the coverslips for 30 min followed by a PBS wash (3X) and the secondary antibody (Alexa Fluor® 488 Donkey anti-Rabbit IgG, product code A21206) were diluted in 10 % FBS at 1:1000 dilution and added to the coverslips for 30 min at RT. Finally, the coverslips had one final PBS wash (3x) and were mounted on glass slides using Vectashield® Antifade mounting media with DAPI (Product code H-1200–10) and sealed using nail varnish.

Slides were imaged using a Zeiss Axio Observer Z1 and LSM 880 Confocal microscope at 40X magnification with oil immersion. Images were taken from 3 different fields of view and a representative image for each treatment is presented within.

### 3.2 Simulating vaping

A549 cells were cultured as described in RPMI supplemented with 10 % FBS, 1 % L-Glutamine, 1 % pen strep. Cells were seeded at 2.5 × 10 ^5^ cells per well into a 35 mm culture dish and incubated overnight at 37° C, 5 % CO_2_ to form a monolayer prior to treatment. A chamber traditionally used for hypoxia studies (Modular Incubator Chamber (MIC-101), Billups-Rothenberg inc, California, US) was used as a sealable unit to permit controlled vapour exposure to the cells to simulate the lung environment. The experimental set up is described in Supplementary Figure 1. To measure primary exposure of cells to vapour, cell culture dishes were placed within the chamber. One end of the tubing was left open, while the other was attached to a vacuum pump (Fisherbrand™ FB70155) with a pumping speed of 9.2 L/min which was comparable to an average adult with a minute ventilation of 6–10 L/min [54], [55]. Furthermore, the chamber used here had a total capacity of ∼6.2 litres (Internal Diameter:∼26.7 cm, Height: 11 cm), which was comparable to the average total lung capacity of an adult being ∼6 litres, which meant this model was suitable for simulating the human lung and average inhalation/exhalation rates [55]. To simulate vaping an Aspire Flexus Q Pod (Aspire, Shenzhen, China), hereafter referred to as END was attached to the open tubing. The END was used according to the manufacturer’s guidance and was suitable for use with the vaping fluids and flavourings used in this study. This END has a built-in safety mechanism to prevent the user from inhaling for more than 6 s at a time. Therefore, for this experiment 6 s puffs were utilised as the worst-case scenario. Although this was out with the CORESTA recommendations for routine analytical machine e-cigarette aerosol generation and collection, which states puff duration should be 3 s ± 0.1 s [56]. In this case it was appropriate to evaluate the puff duration as the design of the END enables the user to have longer duration puffs. Furthermore, several studies have shown that vape users are having longer than 3 s puffs [57], [58]. Five vaping episodes were simulated each with 10 puffs, a minimum of 1 h between vaping episodes. This number of vaping episodes was chosen as it was estimated that young people on average would vape 5 times over 8 h [59]. The cells remained in the chamber overnight at 37° C, 5 % CO_2_ prior to analysis.

Freshly diluted vaping fluid was used for each experiment, using a 1:1 ratio of PG:VG and 10 % Menthol as recommended on the supplier’s website (Dark Star, York, UK). Before commencing the simulation, the END was primed prior to vaping to prevent a “dry-puff”. Once the device was primed the media was changed to fresh complete RPMI and the cells were sealed in the chamber. One tube was connected to the vacuum pump and the END was secured to the second tube. The END was activated by the vacuum pump drawing vapour into the chamber for 6 s, then was detached for 30 s to permit the vapour to diffuse out of the chamber before repeating this process 10 times to mimic inhalation and exhalation. This was in accordance with the recommended standards suggesting 2 puffs per minute [56]. This process was repeated 5 times over an 8 h period to mimic the vaping habits of users as previously described. Cells exposed to the vapour within the chamber were regarded as “primary vapour” and cells that had been placed outside of the chamber at the end of the opened tubes in the incubator were regarded as “secondary vapour”. As they were being exposed to vapour over 24 h as any remaining vapour diffused out from the chamber. Untreated Control (UTC) samples were subjected to the same conditions within the chamber but without the presence of vaping fluid to ensure cell viability was not affected by the chamber or vacuum pump conditions.

Analysis of the cells was performed as previously described, cell viability using nigrosin exclusion, cell death using Annexin/PI FACS flow cytometry and NFκB translocation by immunostaining and imaged by confocal microscopy (Zeiss Axio Observer Z1 with LSM880). The data represented here was performed in triplicate.

### 3.3 Qualitative analysis of vaping fluids

A qualitative scan of the 2 constituent vaping fluids and the 5 flavourings was performed by Institute of Occupational Medicine (IOM), (Edinburgh, UK). Briefly, 10 µL of the sample was diluted in 1 mL of carbon disulphide. An aliquot of each sample was then analysed by GC/MS. The GC was fitted with a 30 m OV-5ms capillary column and programmed to heat from 35 to 340°C. The MS was set to scan from 25 to 350 Daltons every 0.30 s in electron impact ionisation mode. The total ion chromatograms (TICs) generated were examined and the mass spectra of the major peaks compared with reference mass spectra in the U.S. National Institute of Standard & Technology’s mass spectral library

The samples were also analysed for the presence of 45 different metals and other elemental analytes by Inductively Coupled Plasma/Atomic Emission Spectrometry (ICP/AES), (IOM, Edinburgh, UK) and the concentrations provided in µg/mL.

### 3.4 Statistical analysis

Statistical analysis was performed using Graph Pad Prism version 10.3.1 on data sets of triplicate repeats. Data is displayed as means and either a One-way ANOVA using Dunnett’s multiple comparison test or a Two-Way Anova using Tukey’s multiple comparison test was used depending on the data set.

## 4 Results

### 4.1 Cytotoxicity of vaping fluids on A549 cells

**Fig. 1:**
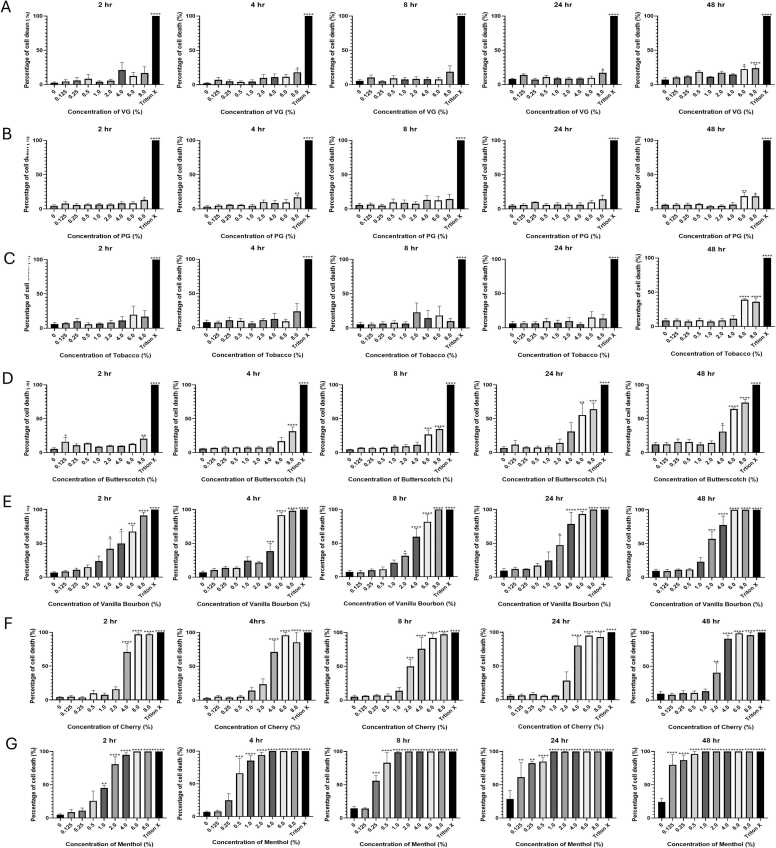
Percentage of cell death (n = 3). A549 cells were treated with A) Vegetable Glycerine (VG), B) Propylene glycol (PG) C) Tobacco, D) Butterscotch, E) Vanilla Bourbon, F) Cherry and G) Menthol at various concentrations (0–8 %) over a period of 48 hr. Cells were harvested at various timepoints for cell counts (2, 4, 8, 24 and 48 hr). The cell counts were performed by either nigrosin staining or Nucleocounter. Triton X (0.25 %) was used as a positive control of cell death and untreated control was 0 %. This was performed in triplicate (n = 3). Error bars represent the standard error of mean (SEM). Statistical analysis was performed using Graph Pad Prism version 10.3.1. An Ordinary One-way Anova with Dunnett’s Multiple Comparisons test was performed to compare each treatment to the untreated control where, *p < 0.05,** p < 0.01,***, p < 0.001 and ****p < 0.0001.

It was clear from the cell counts that the two constituent diluents; VG (Fig. 1**A**) and PG (Fig. 1**B**) had limited effect on cell cytotoxicity, however at the higher concentrations tested (6 and 8 %) there was a significant increase in cell death for both VG and PG at the later time points. This peaked at 26 % (VG) and 19 % (PG) cell death after 48 h exposure which may have been in part due to the osmolality of the carrier substance liquids, rather than from direct toxicity. As the osmolality of 1 M PG is 1000 mOsm/kg, which equates to 7.6 %, and the published osmolality of RPMI is between 260 – 310 mOsm/kg [60], then 8 % PG would potentially result in a doubling of osmolality. This data suggests that any toxicity observed for the e-liquids was therefore likely due to the flavourings and not the constituent diluent liquids.

Tobacco flavouring was found to be the least toxic vaping flavouring based on the cell count data. Only the two highest concentrations (6 and 8 %) were found to significantly increase the percentage of cell death (39 % and 36 % respectively), but this was only after 48 h exposure (p < 0.0001). There was no significant difference found at any of the earlier time points (Fig. 1**C**). In comparison, butterscotch started to show significant levels of cytotoxicity at 8 % after only 2 h incubation (p < 0.01) (Fig. 1**D**). Vanilla Bourbon showed significant levels of toxicity (∼40 %) from as early as 2 h at concentrations of 2 % (p < 0.05) and above this increased to ∼60 % after 48 h at 2 % concentration (p < 0.001) but 100 % of cells were non-viable at 6 and 8 % concentrations **(**Fig. 1**E**). This is a notable result as the manufacturer recommends a 3 % concentration of Vanilla Bourbon (Supplementary Table 1). Interestingly, cherry flavouring had little effect up to 1 % concentration even after 48 h incubation, however the higher concentrations 4 % and over showed above 75 % toxicity after only 2 h exposure (p < 0.0001) (Fig. 1**F**). Finally, menthol flavouring was the most cytotoxic flavouring tested by this measure, resulting in 45 % cell death after only 2 h at 1 % concentration (p < 0.01), by 48 h the majority of cells exposed to menthol flavouring were non-viable even at the lower concentrations tested where more than 80 % of cells were dead at the lowest concentration tested (0.125 %) after 48 h exposure (p < 0.0001) **(**Fig. 1**G**), this was also evident from microscopy where the majority of the cells were rounded up and lifted from the well. The manufacturer recommends a 10 % concentration of menthol to be used in ENDS for the optimal user experience (Supplementary Table 1), which was not tested here as we observed consistent complete cytotoxicity (100 % cell death) from 6 % concentration after only 2 h.

**Fig. 2:**
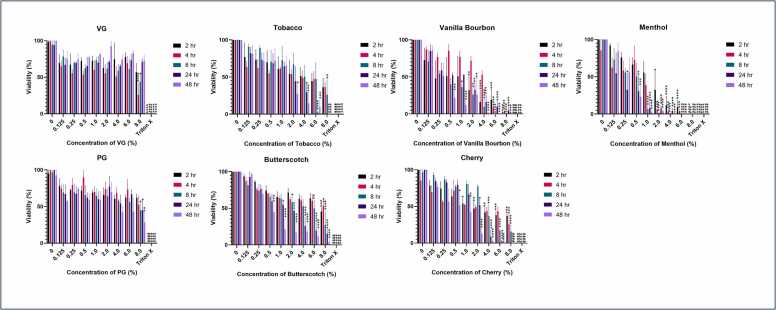
Viability of A549 cells following exposure to vaping fluids over 24 h (n = 3). A549 cells were exposed to various concentrations of different vaping flavourings over a 48 h period. Alamar Blue was used to measure the viability of each of the reagents in isolation. Samples were taken at 2, 4, 8, 24 and 48 hr. This was repeated in triplicate and statistical analysis was performed using Graph Pad Prism version 10.3.1. A Two-way Anova using Tukey’s multiple comparison test was performed to compare each concentration of reagent to their respective untreated control and between timepoints. Statistical significance recorded here is in comparison to the untreated control where, *p < 0.05, **p < 0.01, ***p < 0.001, ****p < 0.0001.

Alamar Blue was used to test the metabolic activity of the lung epithelial cells following incubation with the various vaping flavourings and constituent diluents **(**Fig. 2), a decrease in metabolic activity is associated with poor cell viability. VG and PG both had very little effect on cell viability with only the highest concentration showing any significant effects. Tobacco flavouring was the least cytotoxic with cell viability only appearing to be significantly impacted at the highest concentration tested after 8 h and at concentrations above 2 % and 4 % but only after 48 h (p < 0.05) and 24 h (p < 0.05) respectively. In comparison menthol flavouring is by far the most toxic vaping fluid tested here, with a significant proportion of the cells dead or dying even after only 2 h at concentrations of 2 % and higher (p < 0.05). After 24 h exposure even concentrations as low as 0.25 % were having a significant effect on cell viability with only 32 % remaining viable at this concentration (p < 0.05).

**Fig. 3:**
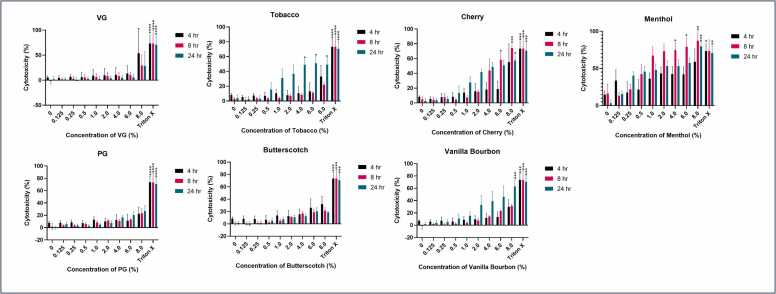
Cytotoxicity of vaping fluids on A549 cells (n = 3). A549 cells were exposed to various concentrations of different flavourings of vaping fluid over a 24 h period. Lactate dehydrogenase (LDH) was detected using a Cyto Tox 96® Non-Radioactive Cytotoxicity Assay (Promega) to measure the cytotoxicity of each of the reagents in isolation. Samples were taken at 4, 8 and 24 hr. This was repeated in triplicate and statistical analysis was performed using Graph Pad Prism version 10.3.1. A Two-way Anova using Tukey’s multiple comparison test was performed to compare each concentration of reagent to their respective untreated control and between timepoints. Statistical significance was recorded here in comparison to untreated control where *p < 0.05, **p < 0.01, ***p < 0.001, ****p < 0.0001.

Since measuring metabolic activity can be variable, and there could be other potential reasons for low metabolic activity other than cell death, we also investigated lactate dehydrogenase (LDH) release. LDH released into the supernatant is suggestive of poor membrane integrity and used as an indicator of declining cellular health. Therefore, increased levels of LDH is indicative of an increased level of cell death [61]. There was little cytotoxicity detected from VG, PG or butterscotch flavouring using this measure, with significance found only with VG at 8 % at 4 h (p < 0.05) when compared to its respective untreated control. However, the error margin for this sample was elevated and may not be a true reflection of the level of cytotoxicity. Menthol appears to be the most cytotoxic flavouring tested. The cytotoxicity peaked at 8 h for most concentrations, reaching ∼80 % cytotoxicity. However, as the half-life of the LDH assay is 9 h the peak could easily have been between 4 and 8 h since the peak may have been attained earlier (Fig. 3).

**Fig. 4:**
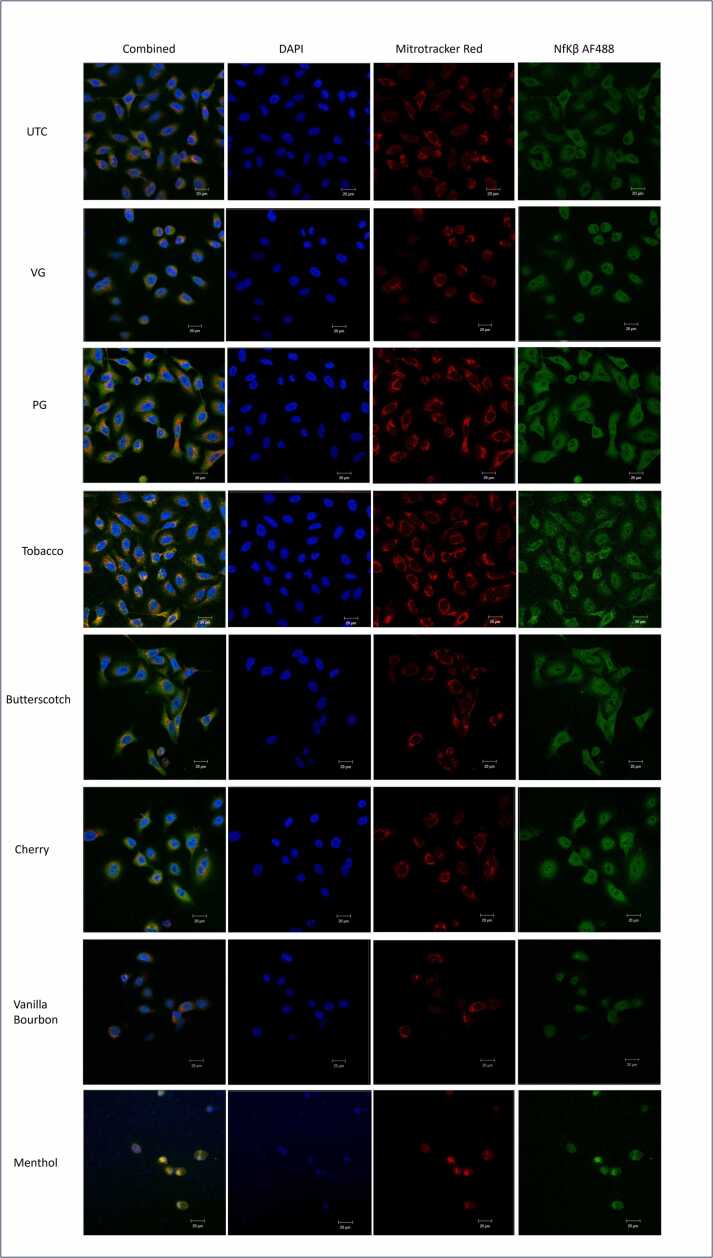
Determining the levels of translocation of NFκB to the nucleus of A549 cells following exposure to vaping fluids (n = 1). A549 cells were treated with Mitotracker® Red prior to incubation with different vaping fluids for 24 h at 37°C, 5 % CO_2_. Cells were fixed with 4 % paraformaldehyde and stained for NFκB with a secondary Alexa Fluor™ 488 antibody. Finally, cells were counterstained with DAPI. Images were captured using a Zeiss Axio Observer Z1 with LSM880 atX40 magnification.

To assess the pro-inflammatory and cell death pathways involved with exposure of A549 cells to vaping fluids immunostaining was performed for NFκB. NFκB translocates to the nucleus as an early indicator of adaptive stress response or apoptosis [53] (Fig. 4). In healthy cells a halo of NFκB staining around an obvious black void (where the nucleus should be) is observed, suggesting that the NFκB is in the cytosol and not the nucleus. Translocation of NFκB to the nucleus was most prominently seen in the menthol flavouring treatment, followed by vanilla bourbon, suggesting that these cells were initiating a cellular stress response and some of the cells were beginning to undergo apoptosis. The other liquid vaping fluids and constituent diluents showed little variation compared to the untreated control after 24 hr.

**Fig. 5:**
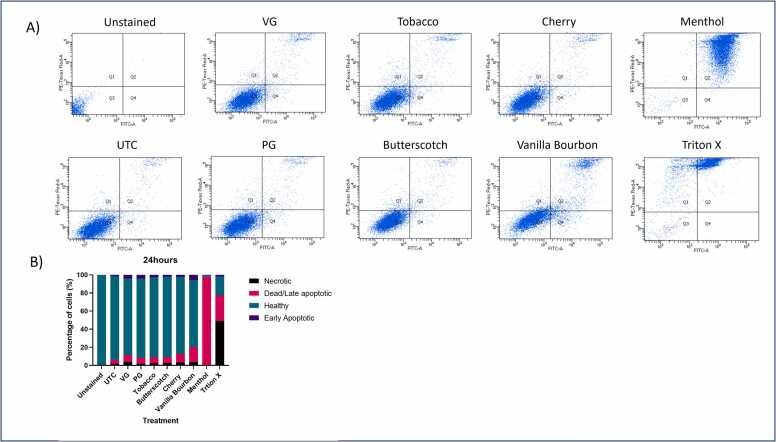
Annexin V and PI staining of A549 cells treated for 24 h (n = 3). A549 cells were treated with different vaping fluids (1 %) over 24 hrs. The cells were stained for Annexin and PI and ran on a BD FACS Celesta. Q1- Necrotic cells (positive for PI) Q2 – Dead or late apoptotic cells (Positive for PI and Annexin) Q3 – Healthy cells (negative for both PI and Annexin) Q4- Early apoptotic cells (positive for Annexin). A) The dot plots for each of the vaping fluids B) Analysis of dot plots. Triton X was used as a positive control of cell death. Data displayed as mean of the 3 replicates.

Cell samples were then analysed using an Annexin/PI kit by flow cytometry to determine the proportion of cells that were early apoptotic, late apoptotic, and necrotic (Fig. 5). The data showed that VG and PG had a slight decrease in cell viability with 7 and 8 % being late apoptotic and both having 4 % of cells early apoptotic compared to the healthy untreated control that showed only 5 % and 1.5 % late apoptotic to early apoptotic, respectively. It was observed that 97 % of A549 cells exposed to menthol vaping fluid (1 %) were late apoptotic, tobacco flavouring again showed the least cytotoxicity with only 10 % late apoptotic and 3 % early apoptotic. Interestingly, VG, cherry and vanilla bourbon had more than double the percentage of necrotic cells compared to the untreated control. There was a significant difference in the number or dead/late apoptotic cells when treated with menthol flavoured vaping fluid compared to the untreated control (p < 0.0001). This data suggests that different flavourings may contribute to different outcomes for lung epithelial cells, and this could be likely due to their different chemical compositions.

## 5 Toxicity of vaping aerosol on A549 cells

From the data acquired here we have demonstrated that menthol vaping flavouring was the most toxic from the range of flavourings tested. To give a more physiological representation of the effects of exposure to the aerosolised vapour, an *in vitro* model was devised (as illustrated in Supplementary Figure 1). The flavouring was heated to 160°C and placed along with A549 cells into a hypoxia chamber. Using a vacuum pump to simulate “puffs” the whole chamber was placed in an incubator with the tubing open to allow for normal gas exchange and incubated for 4, 8 and 24 h at 37°C 5 % CO_2_.

There was no difference observed in cell morphology or amount of cell debris at either 4 or 8 h exposure to primary and secondary vapour. However, by microscopy examination, after 24 h exposure there seemed to be a decrease in the total number of cells and an increase in the amount of cell debris for both primary and secondary vapour compared to their respective untreated controls, suggesting that the menthol vapour could be influencing the lung epithelial cells in this model. Furthermore, immunostaining of NFκB showed slight translocation to the nucleus in the primary and secondary vapour exposed cells as the voids where the nucleus should be, were less defined than those of the untreated controls suggesting some of the cells were undergoing an adaptive stress response or apoptosis (Supplementary Figure 2). Finally, annexin and PI staining measured by FACS, identified the proportion of healthy and dead cells for each treatment and time point. Primary vapour showed the greatest increase in total number of dead cells (necrotic, early and late apoptotic) compared to the untreated controls after 24 h exposure. The profile of cells exposed to the menthol vapour for 24 h was similar to that of the camptothecin control (a known apoptotic inducer) which suggested that the cells were undergoing apoptosis. Another noteworthy result was the increase in the number of necrotic cells identified following exposure of cells to both primary and secondary menthol vapour, with 10.4 % and 9.2 % of cells found to be necrotic respectively, compared to only 2.1 % in the untreated control, suggesting that menthol vapour has the potential to have a significant effect on lung epithelial cells (Supplementary Figure 3).

To further analyse the cytotoxic effect vaping could have on A549 cells, a vaping device was used with 10 % percent menthol flavouring and 1:1 ratio of VG and PG. We demonstrated that heating the vaping fluids to temperatures of ∼160°C with 1 vaping episode of 10 puffs [62] were enough to induce cytotoxic effects, however research has shown that young people vape on average 5 times daily [59] and exclusive experienced e-cigarette users vape a mean of 20 times per day accounting for a total of 200 puffs [63]. Furthermore, it is known that the most common temperature range of vaping devices is 200°C to 250°C and therefore to simulate this effectively, we tested a vaping device with our *in vitro* model. We incorporated 5 vaping episodes of 10 puffs over a period of 8 h to mimic a younger user’s vaping habits [59].

**Fig. 6:**
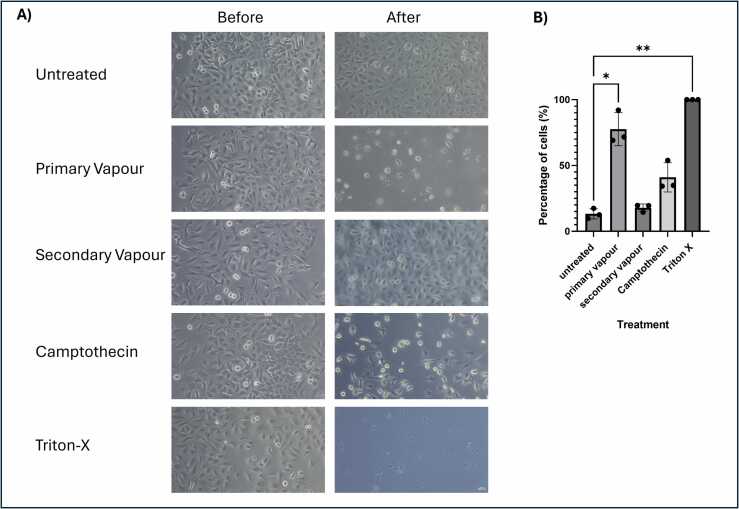
Viability of cells following 5 vaping episodes (n = 3). A549 cells were incubated using the in vitro model described in Supplementary Figure 1 for primary and secondary vapour. Cells were exposed to 10 % Menthol in a 1:1 ratio of VG:PG via an END for 6 s per puff, with a 30 s pause between puffs (total 10 puffs per “episode”) this was repeated 5 times over 8 hourswith a minimum of 1 h between episodes, cells were imaged and harvested the next morning. A) Cells were imaged before and after incubation to observe any morphological changes and cell number B) Cells were stained with nigrosin and counted using a haemocytometer this was repeated in triplicate. Data was displayed as mean percentage death. Camptothecin was used as a positive control of apoptosis and Triton-X was used as a positive control for necrosis. Statistical analysis was performed using a One-way Anova and Dunnett’s multiple comparison test using Graph Pad Prism 10.3.1. Statistical significance was recorded in comparison to the untreated control where *p < 0.05 and **p < 0.01.

We initially observed a significant reduction in the number of viable cells following exposure to the primary vapour when compared to the before images and those of the respective controls (Fig. 6A). Cell viability counts showed a significant increase in the number of dead cells in both samples exposed to primary vapour (∼77 %) (p < 0.05) and treated with Triton X (100 %) (p < 0.01). There was no significant difference observed between secondary vapour exposure and the untreated control (Fig. 6B).

**Fig. 7:**
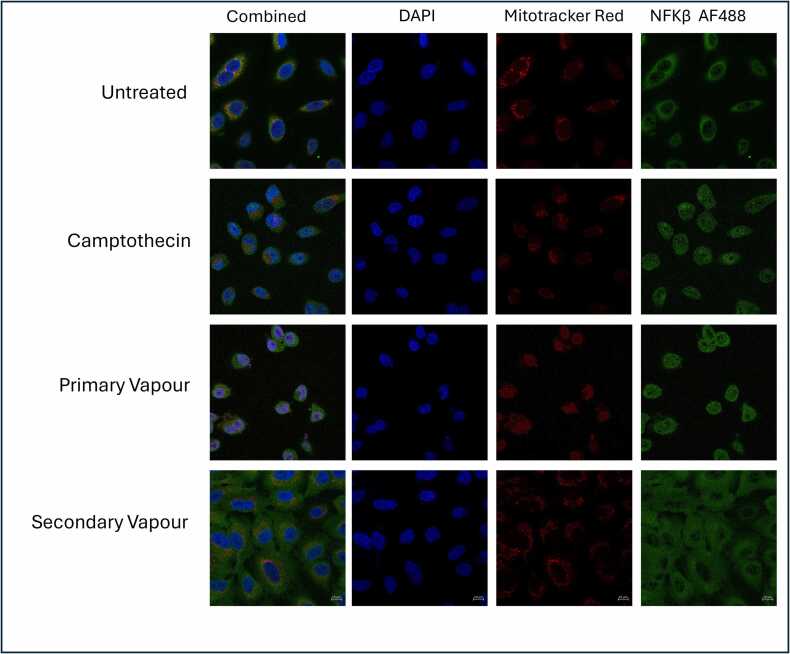
Determining the levels of NFκB translocation to the nucleus of A549 cells following 5 vaping episodes (n = 1). A549 cells were treated with Mitrotracker® Red prior to incubation with 10 % Menthol for 24 h at 37°C, 5 % CO_2_. Cells were fixed with 4 % paraformaldehyde and stained for NFκB with a secondary Alexa Fluor™ 488 antibody. Finally, cells were counterstained with DAPI. Images were captured using a Zeiss Axioport Z1 with LSM880 confocal microscope at X63 magnification. In healthy cells the NFκB should be found in the cytoplasm and form a halo which appears on the images as a dark hole in the middle. As the cells begin to enter apoptosis the NFκB translocates to the nucleus thus losing this halo.

When investigating the levels of NFκB translocation, it was observed that primary vapour was most similar to camptothecin treated cells which is an apoptotic positive control. In both cases the characteristic halo which represents healthy cells is lost as the NFκB translocates to the nucleus as an early indicator of apoptosis (Fig. 7).

**Fig. 8:**
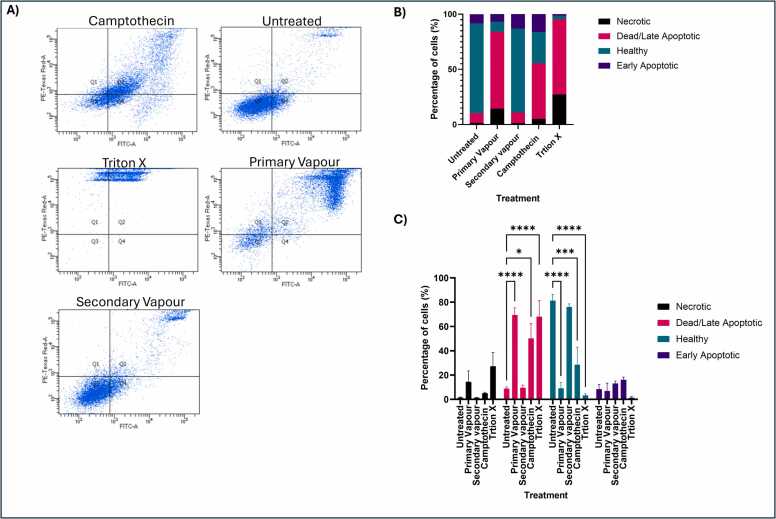
Annexin V and PI staining of A549 cells exposed to vaping (n = 3). A549 cells were incubated using the invitro model described in Supplementary Figure 1 for primary and secondary vapour. Cells were exposed to 10 % Menthol in a 1:1 ratio of VG:PG via an END for 6 s per puff, with a 30 s pause between puffs (total 10 puffs per “episode”) this was repeated 5 times throughout the working day with a minimum of 1 h between episodes, cells were harvested the next morning. **A**) Annexin and PI staining of A549 cells exposed to different treatments. B) Analysis of each treatment. Data displayed proportionally as a mean of the 3 replicatesfor each treatment. C) Analysis of each treatment. Data displayed as a mean of each cell state e.g. necrotic, dead/late apoptotic, healthy and early apoptotic. Statistical analysis was performed using a Two-way Anova and Tukey’s multiple comparison test using Graph Pad Prism 10.3.1. Statistical significance was recorded in comparison to the respective untreated control where, ***p < 0.001 and ****p < 0.0001.

Comparing the proportion of healthy and dead cells using Annexin/PI staining (Fig. 8) demonstrated that primary vapour exposure had the highest percentage of dead or late apoptotic cells (∼70 %) compared to any of the other treatments and was significantly increased compared to that of the untreated control (p < 0.001). Similarly, the proportion of healthy cells was significantly decreased for those exposed to the primary vapour compared to the untreated control (p < 0.001). The profile of the cells exposed to the primary vapour was most similar to that of the Triton X (positive control) for necrosis. Although there were no significant differences found between the secondary vapour exposed cells and the untreated cells there was an increase in the number of early apoptotic cells akin to that of the camptothecin control for apoptosis. This could suggest that over prolonged exposure, the secondary vapour could potentially result in increased levels of cytotoxicity and warrants further investigation.

## 6 Qualitative and quantitative analysis of vaping fluids

Qualitative GC/MS was undertaken to evaluate the chemical composition of the flavouring and carrier liquids as described in the method section. All flavouring liquids were found to contain PG as the main peak **(**Table 1**)**. The chemicals have been identified by similarity to the mass spectral library (SI) and the corresponding CAS number is provided; however, it should be noted that the peak identified may not be the named compound but is likely to be a closely related substance. When screened against the PubChem, European Chemical Agency and IARC databases several chemicals were identified to be respiratory irritants or potential carcinogens. While cyclohexanone derivates (menthol related compounds) are not explicitly carcinogenic, they are considered respiratory irritants and can lead to damage of CNS, liver and kidney [64].

**Table 1:** Qualitative GC/MS was undertaken to evaluate the chemical composition of the flavouring and carrier liquids. Chemicals have been identified by similarity to the mass spectral library (SI) and listed in order of proportional representation (excluding propylene glycol) as determined by the area of the chromatographic peaks. When screened against the PubChem, ECHA, and IARC databases several chemicals were identified respiratory irritants (highlighted in yellow), or potential carcinogens (highlighted in orange).

Quantitative analysis examined potential concentrations of 45 metals and elements present in each vaping fluid samples (Supplementary Table 2). Many of the analytes examined were either absent or below the detection limits. The total amount of each of the metals detected were calculated based on the recommended concentrations of flavourings as detailed on the supplier’s website (see Supplementary Table 1) and assuming that these flavourings were mixed at 1:1 ratio with PG:VG. The data was displayed as ng/day presuming that in a normal day a vape user has 50 puffs (5 ×10 puffs) as was the conditions tested in this study (Table 2). A notable example was arsenic which was detected in all the samples except vanilla bourbon, which could still have contained concentrations that fell below the detection limit (<0.01 µg/mL) **(**Supplementary Table 2**).** However, when mixed with the VG and PG all the samples were found to contain significant concentrations of arsenic (Table 2) due to the content found in the 2 constituent diluents. The menthol mix, under these conditions was found to contain 60 ng of arsenic. Menthol and butterscotch were found to contain 15 and 4.5 ng respectively of barium. Of note, menthol flavouring was also found to contain high concentrations of calcium (equating to 3354 ng/day), magnesium (equating to 1308 ng/day), sodium (equating to 3070.5 ng/day) and potassium (equating to 1080 ng/day) all of which are highly reactive elements and have the potential to create harmful and toxic compounds.

**Table 2:** Metal and elemental analysis. Vaping fluids were tested for the presence of 45 different metals by inductively coupled plasma/atomic emission spectrometry (ICP/AES) (IOM, Edinburgh, UK). The concentrations of each analyte calculated based on the recommended concentrations of each flavouring and a 1:1 ratio of PG:VG (see Supplementary Table 1). The data is displayed as ng per day assuming a low level of vaping of 50 puffs. This is consistent with how other studies have presented similar data as ng/puff [99], [100]). The raw data can be found in Supplementary Table 2.

	ng/day (50 puffs)				
	Tobacco	Butterscotch	Vanilla Bourbon	Cherry	Menthol
Arsenic (As)	60	58.5	58.2	60	60
Barium (Ba)	Trace	4.5	Trace	Trace	15
Calcium (Ca)	201	2314.5	213.6	206.4	3354
Copper (Cu)	Trace	Trace	Trace	0.9	Trace
Iron (Fe)	Trace	Trace	Trace	Trace	6
Indium (In)	3.6	4.5	Trace	1.8	Trace
Potassium (K)	1128	1140	1164	1164	1080
Magnesium (Mg)	28.2	480	32.7	30	1308
Sodium (Na)	1085.7	1889.25	1165.35	1122.15	3070.5
Phosphorus (P)	28.2	124.5	30.9	29.1	27
Ruthenium (Ru)	28.2	28.5	29.1	30	30
Sulphur (S)	Trace	360	Trace	Trace	48
Tantalum (Ta)	Trace	Trace	Trace	Trace	6
Zinc (Zn)	84.6	88.5	89.1	98.2	87

## 7 Discussion

### 7.1 Public health concern

Initially, ENDS were marketed as smoking cessation aids [65], [66]. However, recent health data and media reports have sparked fresh debate about the potential health risks they pose [67], [68]. Rather than focusing on smoking cessation, the current ENDS market has shifted towards promoting a wide variety of flavoured products aimed at expanding the user base, it is estimated that the number of unique e-cigarette products has more than quadrupled from 453 to 2023 in just 1 year from 2021 to 2022[69]. Often this marketing is targeted at young people, many of whom may never have smoked [70]. The data shows that the largest increase in ENDS usage was in 18–25 year-old demographic, with 1 in 5 individuals aged 11–15-years admitting to trying vaping [3], [71]. Additionally, 7.6 % of young people (11–17 years) are reported to be using vaping devices regularly [72].

The toxic effects of nicotine containing vapours from ENDS are well documented and have been found to induce DNA damage in lung, bladder and heart tissue [32], [42], [73]. Multiple studies have examined the toxic effects of nicotine in ENDS and have been found to contribute to both acute and cytotoxic effects including DNA damage in oral epithelial cells [42] and bronchial epithelial cells [15], [73], oxidative stress and inflammatory responses in lung epithelial cells [73], and cardiomyocytes [73]. What this study contributes to is the emerging evidence on the toxicity of the flavourings added to vaping liquids [74], [75], [76], and the effects of exposure to primary and secondary vapours when the e-liquids are heated up. We assessed the toxicity profiles of 5 popular vaping flavours in the UK; tobacco, butterscotch, vanilla bourbon, cherry and menthol, in comparison to two carrier liquids, PG and VG. Through GC/MS analysis, we observed that several of the chemicals detected were highly similar to known respiratory irritants and potentially carcinogenic chemicals and were present in all flavourings tested. Furthermore, by ICP/AES analysis notable concentrations of heavy metals and other elements were also detected. This is the likely cause of the cytotoxic effects observed in this study.

All the flavour mixes (in 1:1 ratio of PG:VG) tested in this study were found to contain 11.6–12 ng of arsenic per vaping episode (10 puffs), in comparison 1 tobacco cigarette is believed to contain ∼110 ng of arsenic [77], so theoretically vaping appears to be 10X less harmful than smoking based on arsenic concentration. However, when considering the data, especially in young people, who are documented to smoke 1 cigarette a day on average [59] and many who have never smoked are taking up vaping. As vaping doesn’t produce the same lingering smoke or strong odours, it is often perceived as more discreet alternative to smoking, possibly leading to more individuals vaping indoors and in vehicles. Additionally, there are reports showing that people tend to vape far more often than they would have ever smoked. In some cases, people have been known to vape as many as 170–200 puffs per day [63], [78], which would mean a total arsenic exposure of 240 ng/day for the latter using the flavouring mixes used in this study. This is more than double that of a single cigarette, this suggests that the use of ENDS has the potential to become a critical health issue.

### 7.2 Cytotoxicity to A549 cells

The two primary vaping carrier liquids VG and PG exhibited minimal cytotoxicity in this study, aligning with previous findings [18], [79], [80]. This suggests that any toxicity was likely attributable to the flavourings rather than the carrier liquids. While PG is a known upper airway irritant [80], [81], [82], this was not investigated further in this study as we used a human epithelial cell line (A549) to model the alveolar Type II pulmonary epithelium. Furthermore, there have been studies that have shown that reactions between the PG and flavour aldehydes can produce harmful acetals with unexpected toxicological effects [36], [50]. Therefore, it is not inconceivable that future research may find increased toxicity when the PG and VG are combined with flavourings.

From all methodologies employed in this study, menthol was found to be the most toxic vaping fluid to A549 cells, showing significant cytotoxicity as early as 2 hr– a finding consistent with other studies linking menthol vapour to increased DNA damage markers [83]. Cherry and vanilla bourbon also demonstrated significant levels of cytotoxicity compared to respective controls exhibiting LD_50_ at only 2 % concentration and vanilla bourbon at 4 % concentration after 8 h of exposure. In contrast, butterscotch and tobacco induced significant cell death, but only at the highest concentrations tested (8 %).

These results are particularly concerning when studies have shown the sweeter flavours increase the appeal of vaping and are more popular amongst ENDS users including adolescents [84], making them more likely to continue vaping long term. This persistence of vaping is associated with increased duration of vaping sessions, thus resulting in longer exposure [59], and as our data suggest potentially increase the risk of pulmonary toxicity.

To further evaluate the effects of menthol, the most toxic flavouring identified in this study, it was important to screen the flavouring against cells as an aerosolised vapour, thus being clinically relevant from an inhalational perspective. In the absence of a bespoke vaping engine, we utilised a re-purposed hypoxia chamber and vacuum pump to analyse both heated and aerosolised vapours drawn across the A549 cells. The VG and PG were combined as 1:1 ratio with 1 % (Supplementary Figures 2 and 3) and 10 % menthol (Fig. 6, Fig. 7, Fig. 8). Analysis by microscopy revealed that after 24 h of primary exposure there were noticeable morphological changes in the A549 cells, including increased amounts of cell debris and rounded up cells. Similarly, exposure to secondary vapour also resulted in minor morphological changes, however, there was very little cellular debris in comparison. In contrast, the respective control samples displayed a healthy monolayer of A549 cells that remained largely unaffected by the chamber conditions, indicating the changes observed were attributable to the menthol vapour exposure (Fig. 6). It was evident that the prolonged exposure to menthol vapour was having a significant effect on the viability of lung epithelial cells, which concurs with previous findings [85]. After only 4 h of direct exposure to heated menthol vapour, there was a significant increase in the number of necrotic, dead/late apoptotic, and early apoptotic cells compared to the untreated control. When simulating vaping with an END with 50 puffs over 8hrs (5 ×10 puff episodes) statistically significant differences in the ratios of healthy, early apoptotic, late apoptotic and necrotic cells were evident in the cells exposed to 10 % Menthol vapour (primary vapour) compared to the untreated control. We found less than 10 % of cells remained healthy when exposed to primary vapour compared to 80 % in the untreated control.

The secondary vapour exposure was simulated to reflect the potential effects on non-vapers in the vicinity of people using vaping devices, in order to determine the indirect impact of vaping. After 24 h of exposure to the menthol vapour there was a reduction by 6 % in the total number of healthy cells compared to the untreated control. Although this decrease was not found to be statistically significant, the reduction in healthy cells was observed across the 3 replicates. There was variation in the proportion of necrotic, dead/late apoptotic and early apoptotic cells, which may have contributed to the lack of statistical significance. Furthermore, the unpredictable and variable nature of vaping fluids when heated in ENDS may have influenced this result [23], [36].

Few studies have investigated the effects of second-hand exposure to vapour from ENDS, and those that do primarily focus on the toxicity of nicotine rather than the toxicity of vaping flavour [86], [87]. It is important to note that for this study the vapour testing data is based on a single simulated vaping episode of 10 “puffs”, which is estimated to be the equivalent of 1 cigarette [62] and the simulating vaping data is based on 5 individual vaping episodes (each of 10 “puffs”) spread over an 8 h period [62]. In both cases following the vaping episodes cells were analysed the next day, this meant that the cells within the chamber had continual exposure to the vapour remnants within the model system. This is likely to be the case within the lungs of vape users as one study has estimated that an average of 6.25 x 10^10^ particles are deposited in the lungs after a single puff of an e-cigarette and the air remaining in the average adult lung after full exhalation (residual volume) being ∼1.2 L [88]. The increase in frequency of vaping will likely have a more pronounced impact on lung health, which warrants further investigation using this model. Furthermore, it would be advantageous in the future to examine the culture media to measure the deposited and dissolved toxicants that potentially will be present following primary and secondary vapour exposure using this model.

Menthol is known to have mildly analgesic and muscle relaxing activity through mild inactivation of voltage gated sodium channels [89], [90]. The cooling sensation is attributed to its effect on the transient receptor cation channel subfamily M member 8 (TRPM8) [91]. The inclusion of menthol in cigarettes was demonstrated to enhance cytotoxicity [92], and although menthol cigarettes were banned in the European Union and the UK in 2020, the United States have yet to follow suit. Further research should focus on usage of flavoured e-liquids and their long- term impact on other organs such as heart, kidney, brain and reproductive system.

## 8 Conclusions and future implications

In alignment with previous studies, the data presented here clearly suggests that the use of flavourings in ENDs has the potential to cause pulmonary and cardiovascular disorders [85], [93], [94], [95], [96]. This raises concerns about future health epidemics and increased burden on the health care providers, particularly as research suggests that prolonged exposure to vaping could result in impaired pulmonary antimicrobial defences [97], leaving individuals more susceptible to infections.

We have demonstrated the toxicity of various flavourings on lung epithelial cells (A549) with notable differences between flavourings. Menthol emerged as the most toxic followed by vanilla bourbon and cherry. While tobacco was the least toxic flavour tested here, this still demonstrated significant levels of cytotoxicity. The long-term effect of vaping remains unclear, and further research is necessary to investigate the impact of flavourings on human health and indeed the environment to better inform users.

From the 1st of June 2025, the UK government implemented a ban on the sale and supply of single-use vaping devices. Additionally, from October 2026, a new vaping duty will be introduced at a flat rate on 10 mL of vaping liquid. Such measures are in an attempt to curb the number of disposable devices contaminating the environment, but also a step towards reducing the number of youths taking up vaping [98].

Undoubtedly, the use of electronic nicotine delivery systems (ENDS) is widely regarded as a safer alternative for individuals seeking to quit tobacco smoking. However, policymakers must take stronger action to prevent non-smokers (particularly the younger demographic) from taking up vaping. This includes introducing stricter regulations on the sale, distribution, and marketing of ENDS, akin those already enforced for conventional tobacco products. A comprehensive regulatory framework should also be adopted to ensuring that manufacturers adhere to consistent safety standards and quality control measures. Additionally, full transparency regarding the ingredients used in flavourings, especially any potentially harmful substances (as detected in this study) is essential. Such measures will empower consumers to make informed decisions and better protect public health.

## Author statement

Fern Findlay-Greene: carried out the practical research, devised the model system, co-wrote and revised the manuscript, Samantha Donnellan: co- recipient of funding, contributed to research plan and assay design, revised the manuscript, Sharron Vass: primary recipient of funding, conceived the plan of research, undertook supervision, co-wrote and revised the manuscript

## CRediT authorship contribution statement

**Fern Findlay-Greene:** Writing – review & editing, Writing – original draft, Visualization, Software, Methodology, Investigation, Formal analysis, Data curation. **Samantha Donnellan:** Writing – review & editing, Visualization, Supervision, Methodology, Formal analysis. **Sharron Vass:** Writing – review & editing, Writing – original draft, Supervision, Project administration, Methodology, Funding acquisition, Formal analysis, Conceptualization.

## Funding

This work was funded by the School of Applied Sciences, Edinburgh Napier University in entirety. The qualitative and quantitative analysis was undertaken as paid work by Institute of Occupation Medicine, Edinburgh.

## Declaration of Competing Interest

The authors declare that they have no known competing financial interests or personal relationships that could have appeared to influence the work reported in this paper.

## Data Availability

Data will be made available on request.

## Footnotes

Appendix A Supplementary data associated with this article can be found in the online version at doi:10.1016/j.toxrep.2025.102092.
