# Supplementary material for: Analysing the acute toxicity of e-cigarette liquids and their vapour on human lung epithelial (A549) cells in vitro
Source: Toxicol Rep. 2025 Jul 18;15:102092. doi: 10.1016/j.toxrep.2025.102092 (PMC12329099; doi:10.1016/j.toxrep.2025.102092)
Supplement: Supplementary file 1 — Supplementary material [file mmc1.docx]

**Supplementary Methods**

**Vapour testing**

To test the toxicity of vapour on A549 cells, cells were first seeded and cultured as previously described. A chamber traditionally used for hypoxia studies (Modular Incubator Chamber (MIC-101), Billups-Rothenberg inc, California, US) was used as a sealable unit to permit controlled vapour exposure to the cells and serve as a surrogate lung.  The experimental set up is described in **Supplementary** **Figure 1**.  Briefly, 1% solution of menthol vaping fluid was diluted in 1:1 ratio of PG and VG, then heated. The 1% menthol concentration was chosen in the first instance because concentrations of 2% and greater all showed significant cell death even after only 2 hr exposure.  Vaping devices have a range of temperature options but are mainly used at 100-315°C. Therefore, it was decided to heat the flavourings to 160°C.  To measure the primary exposure of cells to vapour cell culture dishes were placed around the container containing heated vaping fluid vessel and sealed inside the chamber (**Supplementary** **Figure 1**).  One end of the orange tubing was left open, while the other was attached to a vacuum pump (Fisherbrand^TM^ FB70155) with a pumping speed of 9.2L/min which was comparable to an average adult with a minute ventilation of 6-10L/min (McConnell 2013, Pleil *et al.*, 2021). Furthermore, the chamber used here had a total capacity of ~6.2 litres (Internal Diameter:~26.7cm, Height: 11cm), which was comparable to the average total lung capacity of an adult being ~6 litres, which meant this model was suitable for simulating the human lung and average inhalation/exhalation rates (Pleil *et al.*, 2021).  . To simulate inhalation the vacuum pump was activated and the open end of the second piece of tubing was sealed tightly by hand for 10 s at a time. This process was repeated 10 times, allowing time in between for the vapour to disperse, thus mimicking exhalation. The vacuum pump was then detached, and both sides of the tubing were left open to allow normal gaseous exchange. The cells remained within the chamber and were placed into an incubator at 37°C, 5% CO_2_ and samples were taken at 2, 4 and 24 hr.  To measure passive or secondary exposure of cells to the vapour cell culture dishes were placed next to the open tubing of the chamber in the incubator, which allowed any of the remaining vapour in the chamber to diffuse out. Untreated Control (UTC) samples were treated under to the same conditions within the chamber but without the presence of vaping fluid to ensure cell viability was not affected by the chamber or vacuum pump conditions.  Cell images, Annexin/PI staining and immunostaining were performed as previously described.
