# Supplementary material for: Analysing the acute toxicity of e-cigarette liquids and their vapour on human lung epithelial (A549) cells in vitro
Source: Toxicol Rep. 2025 Jul 18;15:102092. doi: 10.1016/j.toxrep.2025.102092 (PMC12329099; doi:10.1016/j.toxrep.2025.102092)
Supplement: Supplementary file 2 — Supplementary material [file mmc2.pdf]

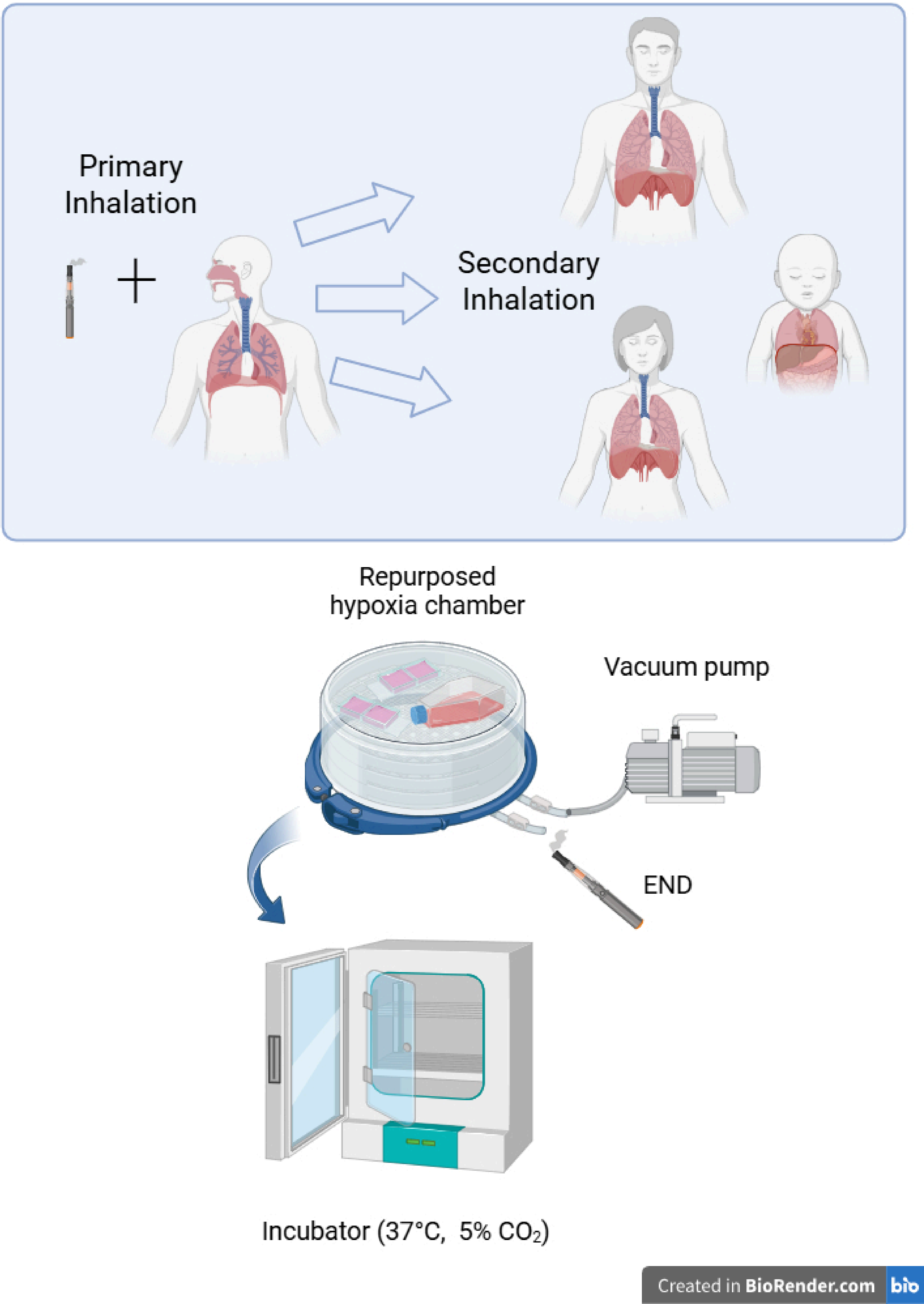

Primary Inhalation
Secondary Inhalation
Repurposed hypoxia chamber
Vacuum pump
END
Incubator (37°C, 5% CO_2)
Created in BioRender.com
