# Supplementary figures and images for: Analysing the acute toxicity of e-cigarette liquids and their vapour on human lung epithelial (A549) cells in vitro
Source: Toxicol Rep. 2025 Jul 18;15:102092. doi: 10.1016/j.toxrep.2025.102092 (PMC12329099; doi:10.1016/j.toxrep.2025.102092)

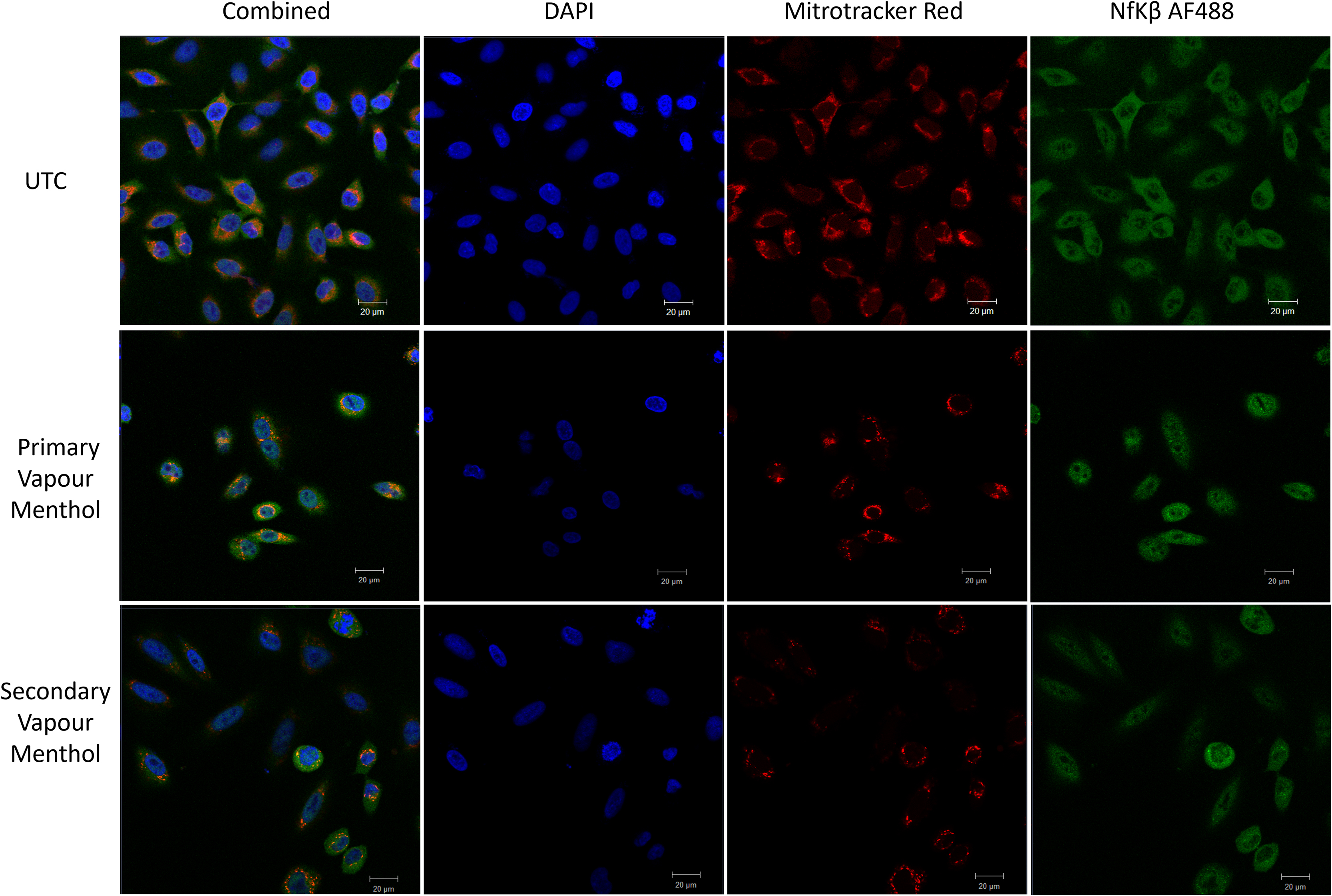

Supplement: Supplementary file 3 — Supplementary material [file mmc3.jpg]

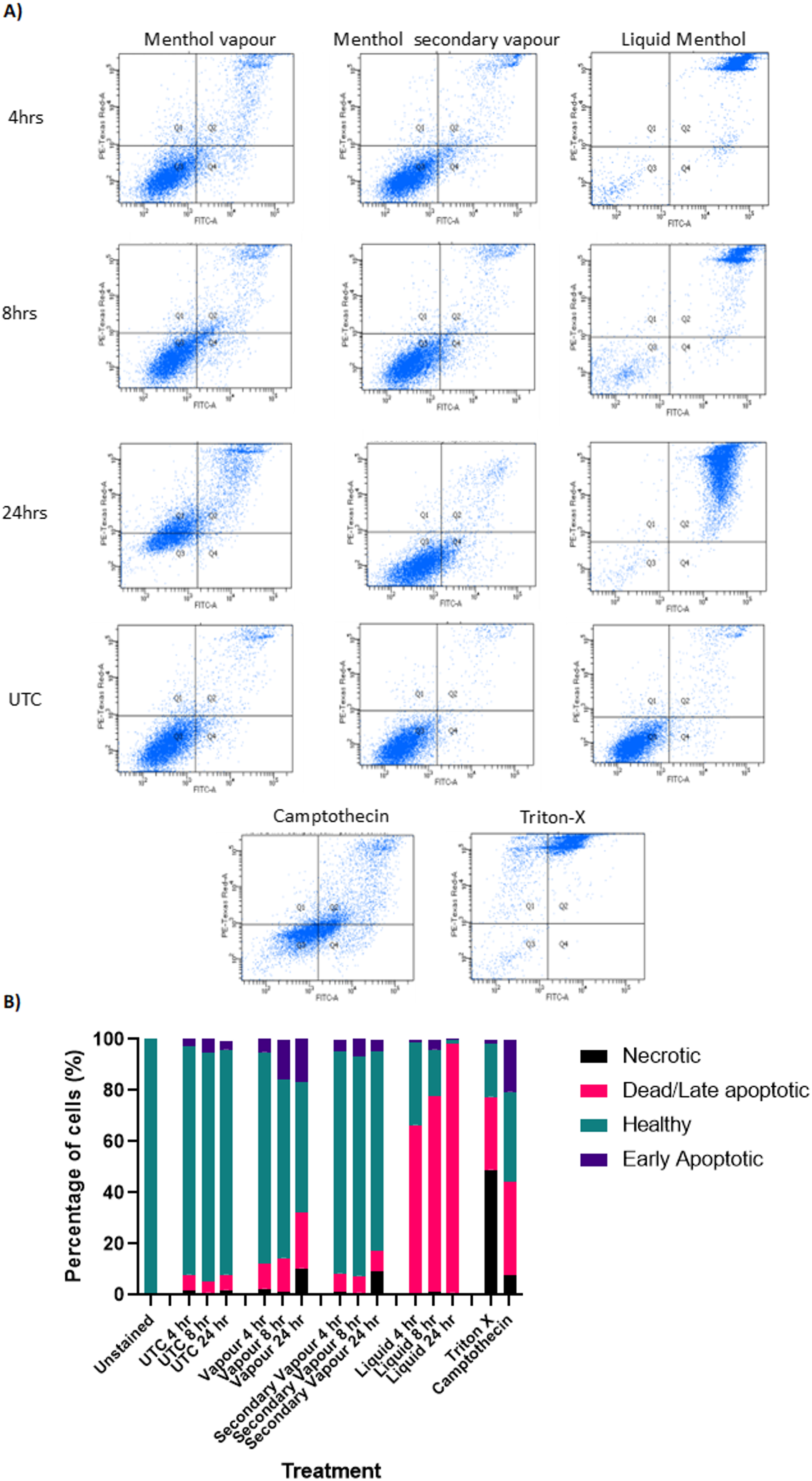

Supplement: Supplementary file 4 — Supplementary material [file mmc4.jpg]

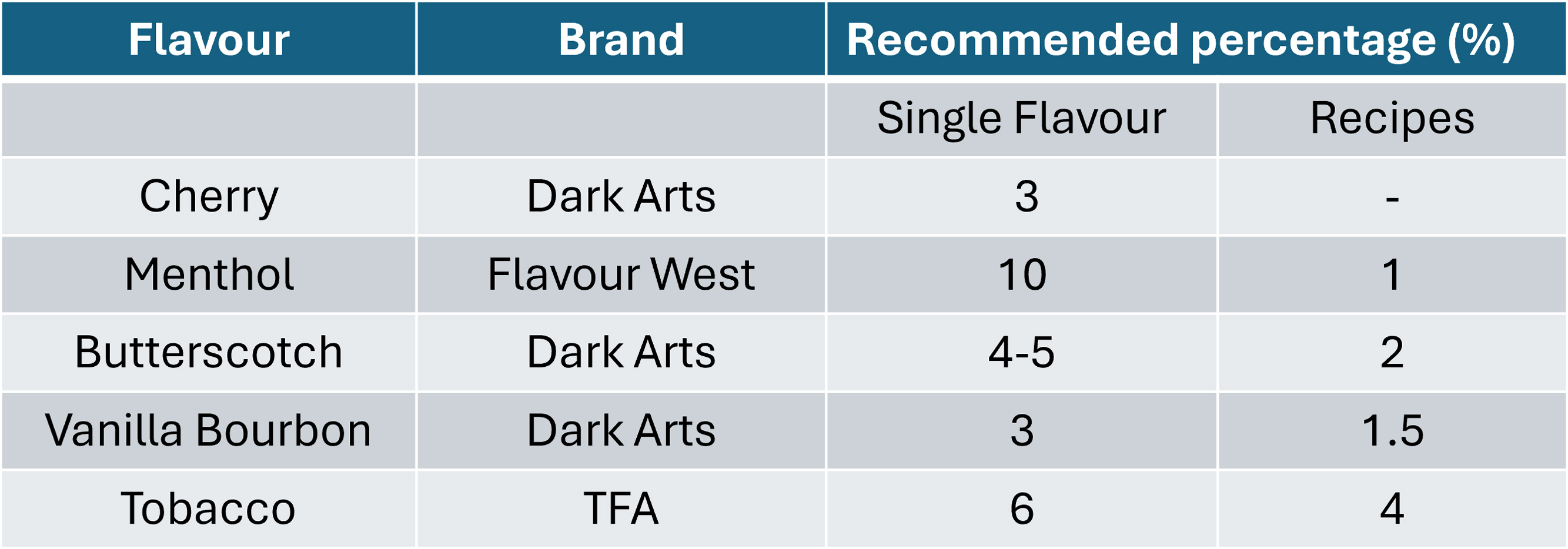

Supplement: Supplementary file 5 — Supplementary material [file mmc5.jpg]

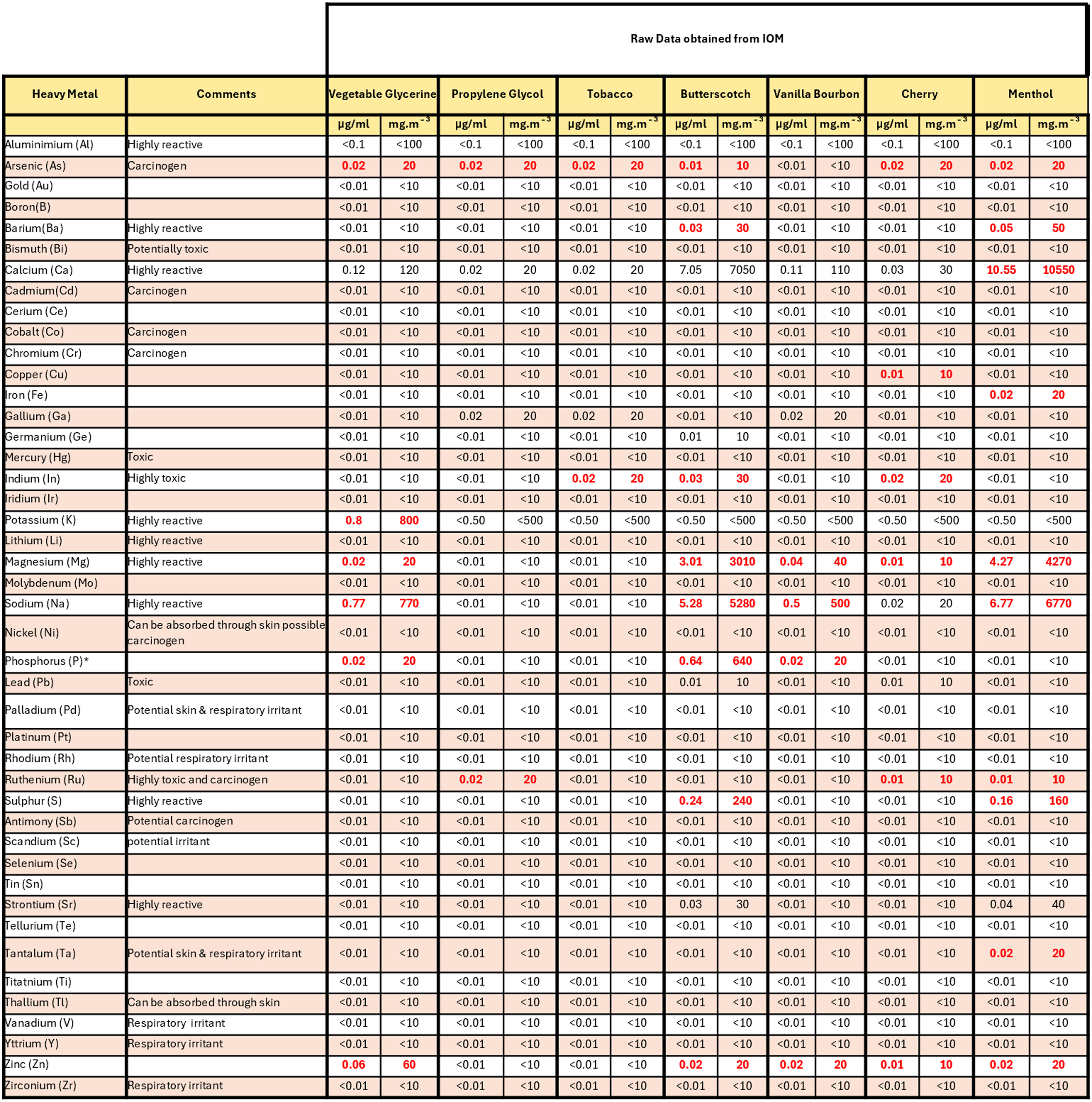

Supplement: Supplementary file 6 — Supplementary material [file mmc6.jpg]
